# Semen quality before cryopreservation and after thawing in 543 patients with testicular cancer

**DOI:** 10.5935/1518-0557.20170009

**Published:** 2017

**Authors:** Antonio MacKenna, Javier Crosby, Cristián Huidobro, Eduardo Correa, Gonzalo Duque

**Affiliations:** 1 Reproductive Medicine Unit, Department of Obstetrics and Gynecology, Clínica Las Condes, Santiago-Chile; 2 Fellow in Obstetrics and Gynecology, Faculty of Medicine, Universidad de Chile; 3 Medical student, Faculty of Medicine, Universidad del Desarrollo, Chile

**Keywords:** Testicular cancer, sperm cryopreservation, sperm banking

## Abstract

**Objective:**

The main objective of this study was to assess semen characteristics of patients with testicular cancer before cryopreservation and after thawing, to evaluate the consequences of this technique on sperm quality in patients with testicular cancer.

**Methods:**

Five hundred eighty-nine samples from 543 patients with testicular cancer were cryopreserved between 1995 and 2015, one aliquot per patient was used for a thawing test to assess the impact of cryopreservation on sperm motility; semen analysis was performed before cryo preservation and after thawing, the result interpretation was carried out using the 2010 World Health Organization (WHO) Laboratory Manual, and consent forms were signed by the patients for freezing and when sperm was used for reproductive purposes.

**Results:**

Hypospermia was observed in 28.7% of samples, the median sperm concentration was 18 million/mL with 35% oligozoospermia; twenty-two patients (4.1%) had azoospermia and 12.7% had severe oligozoospermia, the median sperm count was 31.3 million and 261 semen samples (44.3%) were normal in all parameters according to the WHO; total motile sperm count before cryopreservation and after thawing was 12 (0-412.2) and 7 (0-303.9) million sperm, respectively (*p* < 0.00001, 95% CI 5.48-14.91), which represents a 32% reduction; concerning the utilization of cryopreserved semen samples, only twelve patients (2.2%) used their frozen sperm for reproductive purposes.

**Conclusions:**

An impairment in semen quality was found in almost half of the samples from patients with testicular cancer, only few patients had azoospermia or severe oligozoospermia; sperm cryopreservation significantly reduces sperm motility and total motile sperm count and very few patients use their frozen sperm for reproductive purposes.

## INTRODUCTION

Testicular cancer is a rare tumor, accounting for 1% of malignancies in men worldwide, and its incidence continues to increase in most Caucasian populations, where it is the most commonly diagnosed malignancy in young patients (Shanmugalingham *et al*. 2013). Martinez *et al*. (2015) reported the incidence in Chile to be 7.9/100.000 males (Martinez *et al*., 2015). This cancer affects young adults, with a median age of 32.45 years, and its mortality is only 1.1/100.000 males (Martinez *et al*., 2015), which means that the vast majority of patients with testicular cancer treated will be willing to father a child after the treatment. Testicular tumors impair fertility and, moreover, cancer treatment can adversely affect spermatogenesis because of its effect on the testis (Tournaye *et al*., 2014). Therefore, sperm cryopreservation should be considered as a real option for fertility preservation in males with testicular cancer before receiving any treatment (Garcia *et al*., 2015).

Fertility after testicular cancer treatment is variable and depends on pretreatment semen characteristics, the effect of cryopreservation on the spermatozoa and the consequences of the type of treatment on spermatogenesis (Garcia *et al*., 2015). Sperm banking before cancer treatment enables the subsequent use of semen for Medically Assisted Reproduction (MAR) (Zegers-Hochschild *et al*., 2009), either Intrauterine Insemination (IUI) or Intracytoplasmic Sperm Injection (ICSI), to achieve pregnancy when men are not able to spontaneously impregnate their female partners. However, it has been reported that only 10-15% of males use their cryopreserved samples after cancer treatment (van Casteren *et al*., 2008).

The main objective of this study was to assess semen characteristics of patients with testicular cancer at the time of cryopreservation, before their treatment, and after thawing, to assess the consequences of this technique on sperm quality. A secondary objective was to evaluate the rate of use of cryopreserved sperm after cancer treatment.

## MATERIALS AND METHODS

Five hundred and forty-three patients with testicular cancer were referred from the National Cancer Corporation to the Andrology Laboratory at Clínica Las Condes, Santiago-Chile, for sperm cryopreservation, between 1995 and 2015. After counselling, a consent form, approved by the institutional Research Ethics Committee for sperm cryopreservation, was signed by all men. All semen samples were obtained before surgery and other treatments for testicular cancer and no posterior distinction was made between patients having seminoma and non-seminoma tumors. A total of 589 semen samples were obtained by masturbation into a sterile container and allowed to liquefy for 30 minutes. Semen analysis was performed using standardized procedures throughout the study period, and interpretation of results was carried out using the 2010 World Health Organization (WHO) Laboratory Manual criteria (WHO, 2010).

Semen volume, sperm count and progressive motility were assessed and registered before cryopreservation. Hypospermia (< 1.5 mL), oligozoospermia (< 15 million/mL) and astenozoospermia (< 32% spermatozoa with progressive motility) were defined based on the fifth percentile cut-off, according to WHO definitions (WHO, 2010). Prewashed total motile sperm count (TMSC) was obtained per sample, by multiplying semen volume of the ejaculate by the sperm concentration and the ratio of progressive motile sperm, divided by 100 (Hamilton *et al*., 2015).

Semen samples were cryopreserved in doses of 0.5 to 1 mL aliquots in cryogenic tubes (Nalgene, Fisher Scientific^®^, USA) with Test-Yolk Buffer (TYB^®^, Irvine Scientific, USA) according to the manufacturer's instructions. TYB^®^ contains 20% egg yolk, 12% glycerol and 10µg/mL gentamicin sulfate. One dose per patient was used for a thawing test to assess the impact of cryopreservation on sperm motility. Progressive motility was assessed and registered after thawing and TMSC before and after the procedure was also calculated.

The number of men using their frozen semen for reproductive purposes was recorded as well as the number of patients discarding their samples, either by themselves or as part of a standard procedure after death. No further follow up of patients undergoing fertility treatments could be performed.

Results were described by the Mean ± Standard Deviation for parametric data and by the Median and ranges for non-parametric data. Statistical analysis for differences before cryopreservation and after thawing was performed by the Student t-test for parametric data and by the Mann-Whitney-u test for non-parametric data. A *p*-value lower than 0.01 was considered statistically significant.

## RESULTS

The mean age of the patients was 26.9 ± 6.4 years old and the mean number of cryopreserved doses per patient was 4.9 ± 2.5. Two hundred sixty-one fresh native samples (44.3%) were normal prior to cryopreservation: sperm volume ≥ 1.5 mL, sperm count ≥ 15 million/mL and sperm progressive motility ≥ 32%. The mean number of years of the samples at the sperm bank was 6.6 ± 4.4.

The mean semen volume was 2.2 ± 1.1 mL. The semen volume was ≥ 1.5 mL in 420 samples (71.3%) and < 1,5 mL in 169 samples (hypospermia = 28.7%).

The median sperm concentration was 18 million/mL (0 – 219.5). Twenty-two patients (4.1%) had azoospermia and 69 patients (12.7%) had severe oligozoospermia, defined as ≤ 1 million sperm/mL in native semen. Among samples with spermatozoa, 383 had ≥ 15 million sperm/mL (65%) and 206 had < 15 million/mL (oligozoospermia = 35%). The median total sperm count was 31.3 (0 - 620.2) million. It was ≥ 39 million in 263 samples (44.7%) and < 39 million in 326 samples (55.3%).

The sperm progressive motility in fresh semen was ≥ 32% in 405 samples (68.8%) and < 32 in 184 samples (astenozoospermia = 31.2%). Sperm progressive motility after the thawing test was ≥ 32% in 38.2% of the samples and < 32 in 61.8% of the samples. In six patients no motile sperm was found before cryopreservation and 12 more males did not have motile sperm after the thawing test, yielding a total of 18 patients without motile sperm (3.3%). As shown on table 1, the mean sperm progressive motility before cryopreservation and after thawing was 40.6 ± 19.8 and 25.7 ± 17.2, respectively (*p* < 0.00001, 95% CI 12.82-16.98), which means a 37% reduction in progressive motility.

**Table 1:** Comparison between progressive motility and total motile sperm count before cryopreservation and after thawing in 589 semen samples from 543 patients with testicular cancer.

	BeforeCryopreservation	AfterThawing	Reduction(%)	*p* value
Progressive motility* (%)	40.6 ± 19.8	25.7 ± 17.2	37%	< 0.00001‡
Total Motile Sperm Count † (Millions/mL)	12 (0-412.2)	7 (0-303.9)	32%	< 0.00001§

* Media ± SD, Student t test for comparison before and after

† Median (ranges), Mann Whitney u test for comparison before and after

‡ *p* < 0.00001 (95% CI 12.82-16.98)

§ *p* < 0.00001 (95% CI 5.48-14.91)

The median TMSC before cryopreservation and after thawing was 12 (0-412.2) and 7 (0-303.9) million, respectively (*p* < 0.00001, 95% CI 5.48-14.91), which means a 32% reduction in TMSC (Table 1). Table 2 shows the proportion of TMSC in the ranges ≥ 5, 10-20 and > 20 million before cryopreservation and after thawing.

**Table 2:** Proportion of the samples according to its total motile sperm count before cryopreservation and after thawing.

	< 5	5-20	> 20*
Before cryopreservation†	32.7%	26.5%	40.8%
After thawing†	43.2%	25.9%	30.9%

* Ranges for total motile sperm count, expressed in millions

† Proportion of samples per range

Only 12 patients used their cryopreserved sperm for reproductive purposes, a 2.2% use rate. There is a lack of data about the procedures used and reproductive outcomes in these patients because of a loss of follow up. Nine patients signed a consent form to discard their samples, because they did not need them anymore (spontaneous pregnancies or did not desire children) and 3 samples were discarded because patients were reported dead. The total discard rate was 2.2%.

## DISCUSSION

The mean age of the patients in this study, as in other larger series, confirms that testicular cancer affects young males (Auger *et al*., 2016; Rives *et al*., 2012). This issue and the potential effects of gonadotoxic treatments on semen quality reaffirm how relevant it is to address fertility preservation in this group of patients before cancer treatment.

An impairment in semen quality has been reported in patients with testicular cancer by all large series concerning male fertility preservation before oncology treatment (Auger *et al*., 2016; Rives *et al*., 2012). Indeed, in a recently published study, Auger *et al*. (2016) reported only 50.9 % of men with testicular cancer presenting normozoospermia, using WHO 2010 thresholds. In our study, we had 8.7% of hypospermia, 35% of oligozoospermia and 31.2% of asthenozoospermia. Normozoospermia was observed in 44.3% of samples.

Hypospermia has also been reported by Auger *et al*. (2016) and it can be explained because semen volume depends on mechanisms that are influenced by the level of excitement during semen collection and the psychological condition of cancer patients can influence it.

The median sperm concentration in this study was 18 million/mL with a range from 0 to 219,5, being lower than the median of 30, but the range wider than 16-55 reported by Ragni *et al*. (2003) although, similar to a median of 19.6 million/mL reported by Auger *et al*. (2016). On the other hand, the median number of spermatozoa in the ejaculate in our study was 31.3 million, almost half the median value reported by Auger *et al*. (2016). Indeed, it has been reported that patients with testicular cancer have the lowest sperm concentrations of all oncology categories needing cryopreservation prior to cancer treatment (Bahadur *et al*., 2005; Degl'Innocenti *et al*., 2013). Azoospermia and severe oligozoospermia was found in 4.1% and 12.7% of patients, respectively, which is similar to the values of 6.6% and 15% reported by Rives *et al*. (2012), but lower than the 15.3% azoospermia reported by Ragni *et al*. (2003).

The mean progressive motility was 44.64%, which is similar to the 43% reported by Auger *et al*. (2016) among 2315 samples, but higher than the 35.6% reported by Rives *et al*. (2012) within 320 preorchiectomy samples.

It has been reported that TMSC is the best way to assess sperm quality (Hamilton *et al*., 2015; Borges, 2016). TMSC before cryopreservation was ≥ 5 million in 67.3% of the samples in this study, in contrast with the 59.1% reported by Hotaling *et al*. (2013). Moreover, 40.8% of the samples in our study showed TMSC > 20 million before cryopreservation, which is considered a normal value, with a good prognosis for achieving spontaneous pregnancy (Hamilton *et al*., 2015).

The consequences of cryopreservation on sperm quality are well known. Indeed, spermatozoa can be damaged by the freezing-thawing procedures and sperm motility is the most affected parameter (Degl'Innocenti *et al*., 2013). Comparison of progressive motility before cryopreservation and after thawing showed a significant reduction. As shown on table 1, we found a 37% reduction in progressive motility after thawing, which is like the 30% reported by Hotaling *et al*. (2013). However, when the same analysis was made with the TMSC, we found a 32% reduction, in contrast to the 80% reduction reported by Hotaling *et al*. (2013). This is probably due to a bias in the analysis of the data from the latter study, in which TMSC was expressed as means, not having a normal distribution, and parametric instead of non-parametric tests were used to assess differences between both groups. Indeed, Hallak *et al*. (1999) demonstrated that the behavior of sperm from patients with testicular cancer is the same as that from donors, which means that the reduction of TMSC is proportional to the previous motility in both groups. Degl'Innocenti *et al*. (2013) reported that cryopreserved semen samples with basal number and progressive motility fell below the threshold defined by the WHO (2010), which showed the lowest TMSC after thawing with a bad prognosis. However, only 35% and 31.2% of the samples in this study had oligozoospermia and asthenozoospermia before freezing, respectively. Therefore, the prognosis of cryopreserved sperm in most of the patients with testicular cancer in our study must be considered as good. A recent systematic review reported that 50% of patients that use their cryopreserved samples after cancer treatment achieve pregnancy (Ferrari *et al*., 2016).

It has been reported that TMSC has an accurate prognosis for achieving pregnancy after cryopreservation (Hotaling *et al*., 2013). In our study 67.3% of cryopreserved semen samples had a TMSC ≥ 5 million after thawing (Table 2), which has been previously stablished as the threshold for performing successful IUI (Ombelet *et al*., 2014). Nevertheless, if this procedure is not effective, or if TMSC is < 5 million, almost any cryopreserved semen sample, even when it contains only few motile sperm, could be used for a subsequent infertility treatment with ICSI, having similar reproductive outcomes if compared with the procedure performed with fresh sperm (Borges *et al*., 2007). Only patients without motile sperm would have lower chances to succeed with ICSI (Nagy *et al*., 1998), and this was found in only 18 of the patients included in this study (3.3%), who were properly counselled.

The low number of patients using their cryopreserved sperm for reproductive purposes in this study (2.2%) could be explained because some males recover spermatogenesis and do not need the cryopreserved sperm to impregnate their female partners and, sadly, because other patients succumb to the cancer, which happened only in 3 cases. The use rate in this study was lower than the mean percentage of 7% and 8%, reported for all male cancer patients by van Casteren *et al*. (2008) and Ferrari *et al*. (2016). This issue could be explained because the odds ratio for using frozen sperm in other malignancies compared with testicular cancer is 2.2 (95% CI = 1.1-4.7). The use rate previously reported for patients with testicular cancer is 3.2% (Ragni *et al*., 2003), however, the use rate in our study is even lower. This can be explained by the limited access to MAR in Chile (Mackenna & Zegers-Hochschild, 2014).

Unfortunately, as in other reports, the causes of the low use and discard rates (2.2% and 2.2%) cannot be exactly known in our study, because of the loss of follow up. For that reason, it cannot be concluded that the remaining patients, that still have cryopreserved samples, will need their frozen sperm in the future, although, we can assume that most of them could need it because the mean age of the patients at the time of cryopreservation was only 26.9 years old and the mean number of years of cryopreservation is 6.6 years.

Due to the low use and discard rates, some studies have addressed the cost-effectiveness of sperm banking in male cancer patients, however, if we consider the potential use rate, no conclusions can be obtained yet and more studies, with an accurate follow up, should be performed in the future. In the meantime, sperm cryopreservation for fertility preservation should to be offered to all adult men and post pubertal boys with testicular cancer, before cancer treatment onset, because it is simple, it does not mean a delay in the oncology treatment and is an effective way for fertility preservation.
